# Symbiont evolution during the free-living phase can improve host colonization

**DOI:** 10.1099/mic.0.000756

**Published:** 2019-01-16

**Authors:** William Soto, Michael Travisano, Alexandra Rose Tolleson, Michele Kiyoko Nishiguchi

**Affiliations:** ^1^​ College of William & Mary, Department of Biology, Integrated Science Center Rm 3035, 540 Landrum Dr Williamsburg, VA 23185, USA; ^2^​ Department of Ecology, Evolution, and Behavior, University of Minnesota-Twin Cities, 100 Ecology Building, 1987 Upper Buford Circle, Saint Paul, MN 55108, USA; ^3^​ BioTechnology Institute, University of Minnesota-Twin Cities, 140 Gortner Labs, 1479 Gortner Avenue, St Paul, MN 55108, USA; ^4^​ Department of Biology, New Mexico State University, Box 30001, MSC 3AF, Las Cruces, NM 88003, USA

**Keywords:** host-microbe interactions, ecological diversification, bioluminescence, symbiosis

## Abstract

For micro-organisms cycling between free-living and host-associated stages, where reproduction occurs in both of these lifestyles, an interesting inquiry is whether evolution during the free-living stage can be positively pleiotropic to microbial fitness in a host environment. To address this topic, the squid host *Euprymna tasmanica* and the marine bioluminescent bacterium *Vibrio fischeri* were utilized. Microbial ecological diversification in static liquid microcosms was used to simulate symbiont evolution during the free-living stage. Thirteen genetically distinct *V. fischeri* strains from a broad diversity of ecological sources (e.g. squid light organs, fish light organs and seawater) were examined to see if the results were reproducible in many different genetic settings. Genetic backgrounds that are closely related can be predisposed to considerable differences in how they respond to similar selection pressures. For all strains examined, new mutations with striking and facilitating effects on host colonization arose quickly during microbial evolution in the free-living stage, regardless of the ecological context under consideration for a strain’s genetic background. Microbial evolution outside a host environment promoted host range expansion, improved host colonization for a micro-organism, and diminished the negative correlation between biofilm formation and motility.

## Introduction

Micro-organisms possess the opportunity to adapt to their free-living and host-linked life histories [1]. The extent to which natural selection imposes conflicting demands on microbial genetic variants within these two distinct life histories, possibly even creating fitness tradeoffs, is unclear to evolutionary biologists [2]. Antagonistic pleiotropy has previously been reported between the free-living and host-associated stages in micro-organisms [3, 4]. Even in the absence of antagonistic pleiotropy, if prolonged microbial evolution occurs outside the native host environment, microbial fitness might decrease in a host due to the absence of purifying selection [5, 6]. Conceivably, the greatest opportunity for a micro-organism to adapt and exploit a host–microbe relationship might be when microbial evolution occurs in the host, since this is the environment where beneficial mutations will arise and be promoted by natural selection [7, 8].

As previous work demonstrates, micro-organisms can lose fitness in a host after undergoing evolution in a free-living state [2]. However, much less is known about whether evolution during a free-living existence can increase microbial fitness in a host environment. Numerous scenarios prevail where evolution during the free-living stage could potentially facilitate microbial fitness within a host. First, fitness tradeoffs in microbes could possibly be mitigated by evolution in a non-host environment [9, 10]. Second, microbial fitness within the free-living and host-associated stages might sometimes be governed by positive (synergistic) pleiotropy [11, 12]. Finally, evolution in the free-living stage has important implications for the movement of microbial populations across host adaptive landscapes, where each fitness peak (i.e. fitness optimum) represents local adaptation to a particular host environment or a specific aspect of a host–microbe interaction [13, 14]. For instance, microbial evolution during the free-living stage may permit fitness valleys to be crossed, enabling peak shifts from lower to higher fitness optima within host adaptive landscapes [15–17]. A host–microbe interaction is needed as a model system to explore how evolution during the free-living stage affects microbial fitness in a host.

The mutualism between sepiolid squid (genera *Sepiola* and *Euprymna*) and the marine bioluminescent bacterium *Vibrio fischeri* is a model system for studying associations between bacteria and animal hosts [18]. *V. fischeri* can be grown in pure culture, while sepiolid squid can be raised gnotobiotically [19]. Within the squid host, *V. fischeri* cells reside in a specialized morphological structure called the light organ. The squid utilize light produced by the bacteria for counterillumination [20]. Squid hatchlings emerging from their eggs possess axenic light organs, which are colonized within hours by free-living *V. fischeri* in the ocean. Every day at dawn, squid eject or vent 90–95 % of the light organ bacteria [21]. Bacteria remaining in the squid after venting undergo rapid growth and repopulate the light organ to full capacity by the following evening [22].

**Fig. 1.**
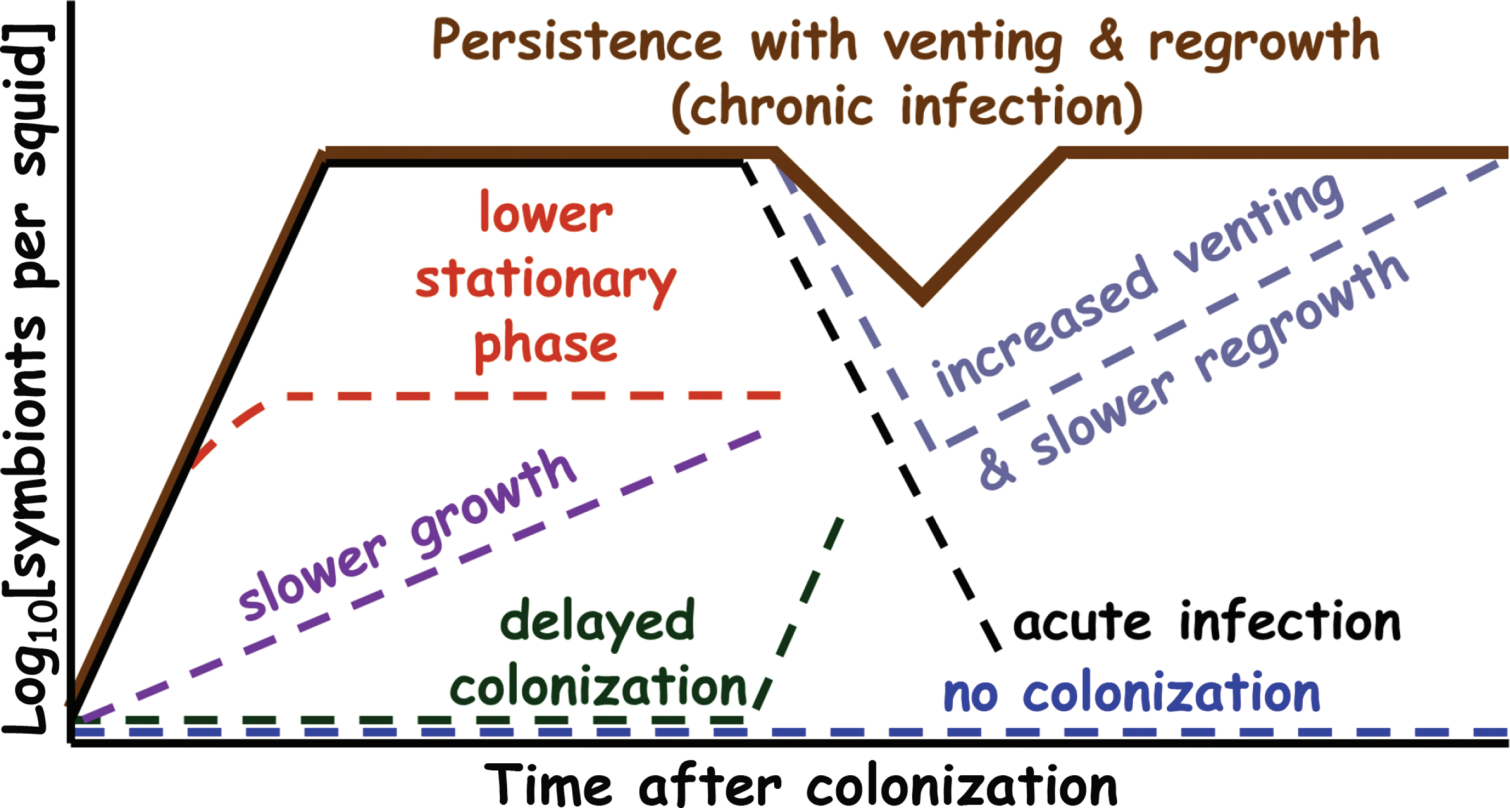
Patterns of squid host colonization by *V. fischeri *. *V. fischeri* isolates collected from the environment (either as a free-living isolate or as a member of a host–microbe interaction) may display a variety of colonization phenotypes in the squid light organ.

When forming light organ symbioses, *V. fischeri* is a host specialist for either sepiolid squid or monocentrid fish [23]. Furthermore, some *V. fischeri* isolates are unable to develop light organ mutualisms with either animal host (they are termed symbiotically incompetent), and are apparently forced to subsist as bacterioplankton or as biofilms attached to abiotic and organismal surfaces [22]. These *V. fischeri* wild isolates range broadly in their ability to colonize squid (Fig. 1) [24]. *V. fischeri* squid specialists establish chronic infections (persistence) in the squid light organ, which becomes densely populated with bacteria (brown line in Fig. 1) [25]. *V. fischeri* fish specialists and symbiotically incompetent strains exhibit deficiencies (broken non-brown lines in Fig. 1) during squid host colonization relative to isolates adapted to cephalopod light organs [22].

In this study, the hypothesis tested was that microbial evolution during the free-living stage can improve host colonization for a micro-organism. In other words, the premise assessed was that microbial adaptation to a non-host environment can be positively pleiotropic to host colonization. The hypothesis was evaluated using static liquid microcosms (unmixed liquid cultures), which are model systems for studying microbial ecological diversification [26]. See the Supplementary Material (available in the online version of this article) for the motivation and justification for using static liquid microcosms to model evolution during the free-living phase for *V. fischeri *. Static liquid microcosms permit environmental heterogeneities and vacant niches to develop that ultimately facilitate microbial adaptive radiations. Liquid microcosms with continuous and intense mixing lack sufficient ecological opportunity for microbial diversification to occur [27]. In a static liquid microcosm, an initial clonal population differentiates into a polymorphic one comprising alternative ecotypes (cell subtypes or varieties occupying different ecological niches), which form different colony phenotypes on solid media [27]. Closely related bacterial strains that vary in colony morphology often colonize hosts quite differently [28]. *V. fischeri* ecological diversification in static liquid microcosms was used to simulate evolution during the free-living stage. The derived *V. fischeri* populations were then compared to the ancestor in their capacity to colonize squid hosts.

Thirteen genetically distinct *V. fischeri* strains from different ecological sources (e.g. squid light organs, fish light organs and seawater) were used, which had a range of capacities for squid host colonization – chronic infections, acute infections and complete failure to initiate any host colonization (Fig. 1) [23]. By utilizing several different strains, this study investigated how microbial fitness inside the squid host would improve for different bacterial genetic backgrounds after evolution during the free-living stage. Microbes with closely related genetic backgrounds can be predisposed to major differences in how they respond to similar selection pressures [29]. This study demonstrates that microbial evolution during the free-living stage (via ecological diversification and biofilm evolution) can generate exaptations for host–microbe interactions. Although the conclusions reported here are for a mutualism, the results in the current study may be applicable to host–pathogen interactions in a more general sense.

## Methods

### Strains, culture media and culture maintenance

Thirteen genetically distinct strains of *V. fischeri* that all form smooth colonies on agar (BeanTown Chemical BT121155, Hudson, NH, USA) plates were studied. The strain characteristics are listed in Table 1. Bacteria were grown in modified seawater tryptone [MSWT; 1.0 % w/v tryptone, 0.5 % w/v yeast extract, 0.3 % w/v glycerol, 513.3 mM NaCl, 50.0 mM MgSO_4_, 10.0 mM CaCl_2_, 10.0 mM KCl, 0.01 mM FeSO_4_, 10.0 mM NH_4_Cl, 0.33 mM K_2_HPO_4_ and 50.0 mM Tris (pH 7.5)]. For enumeration and streaking for isolation, MSWT in 2.0 % w/v agar was used. MSWT in 0.5 % w/v agar was used for swarm motility assays (see below). All liquid cultures consisted of 10 ml MSWT in 25×150 mm borosilicate glass test tubes, including for biofilm assays (see below). The incubation temperature of all liquid and plate cultures was 28 °C. Culture stocks were maintained in an −80 °C freezer in cryovial tubes with cryoprotectant solution [final concentrations: 3.44 M glycerol, 513.3 mM NaCl and 50 mM Tris (pH 7.5)].

**Table 1.** *
V. fischeri* strains used in the study. All strains were the SM morphotype. The strains were described in previous works [18, 24]

**Strain**	**Source**	**Niche**	**Location**	**Squid** **host** **colonization phenotype**
SA1G	*Sepiola affinis*	Squid host	Banyuls sur Mer, France	Wild-type
SR5	*Sepiola robusta*	Squid host	Banyuls sur Mer, France	Wild-type
SI1D	*Sepiola intermedia*	Squid host	Banyuls sur Mer, France	Wild-type
ES114	*Euprymna scolopes*	Squid host	Kaneohe Bay, Hawaii, USA	Wild-type
EM17	*Euprymna morsei*	Squid host	Tokyo Bay, Japan	Wild-type
EB12	*Euprymna berryi*	Squid host	Tosa Bay, Japan	Wild-type
ET00-3-20	*Euprymna tasmanica*	Squid host	Sydney, New South Wales, Australia	Wild-type
ET00-7-1	*Euprymna tasmanica*	Squid host	Sydney, New South Wales, Australia	Wild-type
ATCC 7744	Dead squid	Saprophyte	Coast of Massachusetts, USA	Acute infection, slower growth, increased venting with slower regrowth, lower stationary phase
CG101	*Cleidopus gloriamaris*	Fish host	Townsville, Queensland, Australia	Chronic infection (persistence), lower stationary phase
MJ1	*Monocentris japonicus*	Fish host	Southeastern coast of Tokyo, Japan	Acute infection, lower stationary phase, no regrowth after venting
MDR7	Seawater	Bacterioplankton	Marina del Rey, California, USA	No colonization
WH4	Seawater	Bacterioplankton	Woods Hole, Massachusetts, USA	Acute infection, slower growth, increased venting slower regrowth, lower stationary phase

### Ecological diversification experiments

To create starter cultures, single colonies from all strains were inoculated as pure cultures (monocultures) in test tubes with MSWT and incubated for 12 h at 200 r.p.m. To physiologically acclimate all the strains, these initial cultures were diluted 1/100 into new test tubes containing fresh MSWT media and incubated for another 12 h at 200 r.p.m. From these second cultures, replicate MSWT pure cultures were established with an initial cell density of 5×10^5^ cells ml^−1^ for all strains. One set of replicate MSWT tube cultures was allowed to shake at 200 r.p.m. for 22 days, while another set was allowed to stand (non-shaking) for the same amount of time. From the shaking and non-shaking sets, five replicate MSWT test tube cultures (*n*=5) were destructively sampled every 48 h. MSWT test tube cultures were vortexed with sterile 3 mm borosilicate glass beads (Sigma Z143928, St Louis, MO, USA), serially diluted and plated onto MSWT agar plates. Plate cultures were incubated for 48–72 h. Colonies were classified according to colony morphotype with a stereo microscope, and then the frequencies of smooth (SM) and wrinkly spreader (WS) colonies were determined. Due to an unexpected nonculturability observed in *V. fischeri *, subsequent experiments became necessary. MSWT test tube cultures were shaken over 48 h. These cultures were destructively sampled (*n*=3) every hour to generate plate counts.

The relative diversity (*J′*) of colony morphotypes was calculated using the Shannon–Weaver index and *H′*_max_ [27, 30]. From each of the different time points of this experiment, SM and WS colonies from all strains were randomly isolated from agar plates, grown in test tube liquid cultures for 12 h and preserved at −80 °C as mentioned earlier. For all remaining experiments, *V. fischeri* WS colonies were used from day 22, while *V. fischeri* SM colonies were from the original ancestor. Day 22 was selected based on preliminary experiments. By day 22, the diversification process had reached an equilibrium. SM and WS colony morphotypes were selected randomly from agar plates, and independent isolates were used for each experimental study.

### Antibiotic resistance markers, true-breeding nature of colony morphotypes and growth characteristics of morphotypes

Resistance to chloramphenicol and kanamycin were the markers used in this study. The routine insertion of chloramphenicol and kanamycin resistance genes as neutral markers into the *V. fischeri* chromosome using the mini-Tn7 transposon, respectively mini-Tn7camr and mini-Tn7kanr [31], has already been described in great detail elsewhere [25, 31]. The kanamycin and chloramphenicol resistance markers inserted into the *V. fischeri* strains had no significant effects on bacterial fitness in any of the *in vitro* and *in vivo* conditions examined in this study, which is consistent with earlier work (data not shown, [18, 25]). For all strains, both colony morphotypes (SM and WS) were also streaked for isolation to assess true breeding. For each strain, single SM and WS colonies were separately inoculated into standing test tubes with 10 ml MSWT and incubated for 48 h to determine the resulting growth characteristics (planktonic versus pellicle).

### Pellicle tensile strength, biofilm formation, motility assays and linear regressions

SM and WS morphotypes isolated from ecological diversification experiments were further characterized for pellicle tensile strength (*n*=5), biofilm formation (*n*=20) and swarm motility (*n*=5) assays in MSWT. For the current study, biofilm formation does not include the pellicle that forms further out from the air–liquid (A–L) interface (meniscus). After inoculation, the initial cell density for the pellicle tensile strength and biofilm assays was 5×10^5^ cells ml^−1^. Crystal violet biofilm and motility assays with 0.5 % agar have been described in great detail elsewhere [25]. However, 25×150 mm glass test tubes (with 48 h incubation) were used instead of microwell plates to avoid measuring biofilm formation as a correlated response. Other modifications in the biofilm assays included MSWT replacing SWT and 10 ml volumes for the washes, rinses and resuspensions. After the appropriate washing, rinsing and removal of excess crystal violet stain [25], sterile 3 mm borosilicate glass beads (Sigma Z143928) were added to the otherwise empty test tubes containing the crystal violet-stained biofilms, along with the addition of 10 ml of 100 % ethanol. The test tube biofilms were then vortexed to redissolve and resuspend the crystal violet-stained biofilms in ethanol to allow quantification of biofilm growth with absorbance (600 nm) readings using a spectrophotometer. Test tubes containing uninoculated liquid media were used as negative controls.

For biofilm assays, culture tubes were inoculated with either SM or WS and incubated for 48 h without shaking. Biofilm measurements in test tubes permitted direct model II linear regression analysis for SM motility versus SM biofilm formation and WS motility versus WS biofilm formation with geometric mean statistical considerations for comparisons of slopes with type 1 *α* error=0.05 [30, 32–34]. For pellicle tensile strength assays, culture tubes were inoculated with either SM or WS and incubated for 48 h without shaking. Afterward, 1 mm borosilicate glass beads (Sigma, Z273619) were added to culture tubes to quantify how much total mass could be held by pellicles that may have formed before rupturing [35, 36]. Each 1 mm glass bead had a mass of 0.002 g. The least significant difference with a modified Bonferroni correction using the Dunn–Šidák method (experiment-wise type 1 *α* error=0.05) for all possible pairwise comparisons was calculated separately for the biofilm, motility assays and pellicle tensile strength assays [32]. Standard conventions were followed for *P*-values, namely *ns*=*P*>0.05 (not significant), *=0.01 < *P*≤0.05, **=0.001 <*P*≤0.01 and ***=*P*≤0.001 [32].

### Invasion experiments

For all strains, the ability of the colony phenotypes (SM and WS) to invade when rare against the more common variety was investigated in static liquid microcosms. The combined initial cell density was 5×10^5^ cells ml^−1^ for all competitions. The ratio of the competing colony phenotypes was 100 : 1, and populations were competed for 14 days. Pilot experiments conducted beforehand showed the competitions typically reached an equilibrium within 2 weeks. Antibiotic resistance markers made with mini-Tn7 (discussed earlier) permitted distinction between invasion (natural selection) of the initially rare colony morphotype and phenotypically visible diversification (mutation) in the more common variety. These experiments helped to determine the role of competition as a driver of ecological diversification and whether tradeoffs existed between SM and WS. Two-tailed *t-*tests were conducted for significance (type 1 α error=0.05).

### Animal experiments

Much of the methodology for the squid colonization experiments with *V. fischeri* was described in great detail earlier [18, 37]. Briefly, *Euprymna tasmanica* hatchlings just emerging from their eggs and possessing axenic light organs were used for all experiments. *E. tasmanica* hatchlings were placed in 10 ml scintillation vials with 5.0 ml 34 p.p.t. artificial seawater (Instant Ocean, SS15-10, Blacksburg, VA, USA) and maintained in a 12 h:12 h dark/light cycle at 25 °C. Fresh artificial seawater was changed every 12 h. For each treatment, several squid hatchlings were simultaneously inoculated with 1×10^3^ *V. fischeri* c.f.u. ml^−1^ for monoculture and 50 : 50 competition experiments. This cell density was plentiful enough to guarantee hatchling inoculation with *V. fischeri *. Monoculture experiments were animals solely inoculated with either the ancestral SM or the derived WS but not both. The 50 : 50 competition experiments involved animals simultaneously inoculated with equal numbers of ancestral SM and derived WS. After a 3 h incubation with *V. fischeri *, animals were rinsed three times with sterile seawater to synchronize symbiont colonization within this time window. Animals placed in artificial seawater with no symbionts for 3 h before being rinsed served as negative controls. Animals (*n*=5) were sacrificed every 2 h over a 36 h period to obtain *V. fischeri* squid host colonization data.

Prior to the animals being sacrificed, hatchlings were placed in a luminometer (Turner Designs 20/20, San Jose, CA, USA) to measure bioluminescence [as relative light units (RLUs)]. When the animals were sacrificed, squid light organs were homogenized, serially diluted and plated onto MSWT agar plates for enumeration with or without the presence of chloramphenicol and/or kanamycin [31]. Plate counts of *V. fischeri* SM and WS morphotypes were determined. For statistical analyses, *V. fischeri* isolates were grouped into either ‘all strains’, ‘*Euprymna*’, ‘*Sepiola*’, ‘free-living’ or ‘fish’ strains (Table 1). *V. fischeri* isolates were classified as ‘free-living’ strains (* V. fischeri* ATCC 7744, MDR7 and WH4) if and only if they failed to establish a persistent infection in the squid host and were not isolated from an animal light organ. Strains were also analysed individually. For the *in vivo* studies, the least significant difference with a modified Bonferroni correction using the Dunn–Šidák method (experiment-wise type 1 *α* error=0.05) was calculated for all possible pairwise comparisons [32].

## Results

### Ecological diversification and biofilm evolution generate *V. fischeri* WS from an SM ancestor

The non-shaking test tubes provided niche heterogeneity, which created ecological opportunity that engendered microbial adaptive radiation [27]. After 6–10 days, a pellicle (an A–L interface biofilm) formed at the A–L interface [38, 39]. Two niches now existed in each *V. fischeri* static liquid microcosm comprising ‘water column’ and ‘pellicle’ cells that formed SM and WS colonies on agar plates, respectively. Through mutation and natural selection, WS-derived cells in the pellicle arose from the ancestral planktonic SM cells. The cell densities of the SM and WS morphotypes were tracked over 22 days through plate counts. Fig. S1a shows the cell densities for the SM and WS morphotypes when averaged for all strains. WS colonies in the static liquid microcosms were first detected after about a week of incubation, but the exact time ecological diversification began was strain-dependent. As the WS morphotype’s cell density increased and reached its maximum, the SM morphotype’s population size decreased but did not go extinct. Instead, the SM and WS morphotypes reached different population sizes or carrying capacities, enabling coexistence through 22 days. Relative diversity (*J′*) was calculated and averaged for all *V. fischeri* strains to characterize the coexistence of the SM and WS morphotypes through 22 days in the static liquid microcosms (Fig. S1b). Relative diversity first began to rise with the appearance of the WS morphotype, ultimately reaching equilibrium.

Shaking test tubes served as negative controls, since the constant and vigorous agitation minimizes niche heterogeneity. For all strains, disturbance (shaking test tubes) prevented the generation of diversity, as the disturbed *V. fischeri* populations became completely nonculturable on agar plates within 2 or 3 days (Fig. S1c). As a result, there was no opportunity to characterize the relative diversity of SM and WS in the shaking test tubes. Nonculturability was described previously for *V. fischeri* [28].

### SM and WS are pure lines and distinct ecotypes

Antibiotic resistance markers were used in *V. fischeri* in exactly the same manner as pantothenic acid auxotrophy was in *Pseudomonas fluorescens* [27]. The spontaneous mutation rate of chloramphenicol resistance was negligible in *V. fischeri* (∼1×10^−10^ c.f.u. ml^−1^) and no reversion of the marker phenotype to wild-type was observed. When SM and WS morphotypes were streaked for isolation, only SM and WS colonies arose, respectively, demonstrating that the SM and WS phenotypes were true breeding (Fig. S2a–f). Additionally, antibiotic resistance markers (chloramphenicol and kanamycin resistance) independently confirmed that the SM and WS morphotypes were indeed pure lines with 100 % phenotype penetrance. No phenotypic plasticity was evident with colony formation. SM colonies only and always gave rise to SM ones, while WS colonies only and always gave rise to WS ones. SM colonies formed cultures that exclusively were composed of water column or planktonic cells (with no pellicles present); WS colonies generated cultures containing only pellicles (with no visible water column turbidities present; Fig. S2g). Since *V. fischeri* SM and WS morphotypes, respectively, only gave rise to planktonic and pellicle populations, the *V. fischeri* SM and WS morphotypes in this study were each considered to be unique ecotypes in an analogous manner to *P. fluorescens* [27].

### WS has stronger biofilms, higher pellicle tensile strength and lower motility than SM

A pellicle is a biofilm that forms at the interface between air and a liquid (A–L interface biofilm) [38, 39]. *V. fischeri* WS cultures containing a 5-day-old pellicle were found to be capable of holding back the remaining liquid when the test tubes were inverted or positioned upside down (Fig. S3a, b). Pellicles formed after only 48 h of incubation were not as durable, but their tensile strength was still quantified for statistical considerations by measuring how many glass beads were added before the pellicle ruptured (Fig. S3c–e). The longer the pellicle was allowed to incubate (i.e. ‘age’ or ‘mature’), the more resistant it became to being ruptured by the collective weight of glass beads (Fig. S3d). For all strains, *V. fischeri* WS had greater tensile strength than *V. fischeri* SM (Fig. S3e). Regardless of which strain was being considered, the *V. fischeri* SM ecotype did not form a pellicle within 48 h of incubation at 28 °C. As a result, the SM ecotype’s tensile strength for each strain was essentially zero. For all strains, the WS ecotype displayed higher biofilm formation (adherence to glass) than the SM ecotype (Fig. S4a), while the SM ecotype possessed greater motility than the WS (Fig. S4b).

**Fig. 2.**
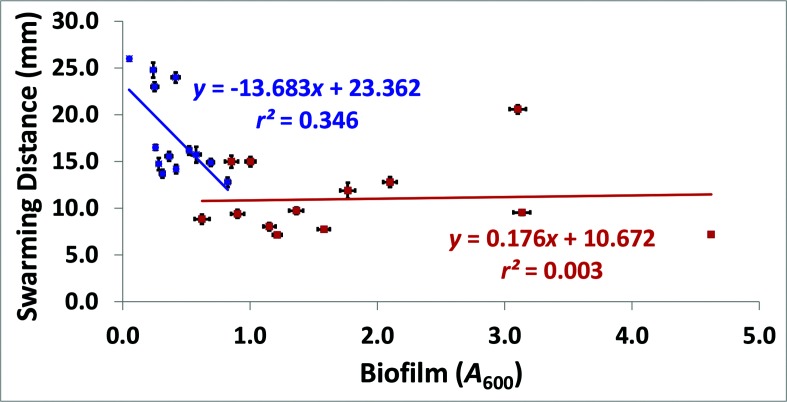
The relationship between motility and biofilm formation in WS and SM. The WS (orange) relationship between motility and biofilm formation lost the negative correlation initially present in the SM (blue) association between these two traits. Each point represents a value for one strain. The error bars represent the standard error of the mean.

A notable change in the linear relationship between motility and biofilm formation occurred as a result of ecological diversification, when all 13 *V. fischeri* strains were included in the analysis (Fig. 2). The ancestral association SM motility versus SM biofilm had a negative linear relationship that was significant [*F*_2(11,11)_=3.85(*)]. The derived regression WS motility versus WS biofilm did not exhibit a linear relationship that was significant [*F*_2(11,11)_=1.12 (ns)]. The antecedent regression coefficient (*b*_ancestral_=−13.683) of SM motility versus SM biofilm was significantly different from the derived slope of WS motility versus WS biofilm (*b*_derived_=0.176). This result indicated that the ancestral link between motility and biofilm formation had changed in *V. fischeri* as a result of microbial adaptive radiation in static liquid microcosms [Imbrie test *T*_12_,_2(13)_=5.040(***)] (Fig. 2). For many strains, the SM ecotypes were at least able to form weak crystal violet biofilms to a significantly greater extent than the negative control (Fig. S4a) [30, 32–34, 40]. A significant positive linear regression was noted between the total mass of 1 mm glass beads sustained before rupturing and biofilm formation for *V. fischeri* WS (*F*_2(11,11)_=71.99***, Fig. S4c). A similar analysis for *V. fischeri* SM was not possible, since no SM ecotype of any strain formed a pellicle.

### Negative frequency-dependent selection operates between WS and SM

A fitness value of 1.00 (the ratio of the Malthusian parameters of the initially rare ecotype to the common one over 2 weeks) indicates equal competitive ability between two contestants [27]. For all strains, each colony morphotype was able to invade against the opposing, more numerous, counterpart when rare, suggesting that a competitive tradeoff existed that was negatively frequency-dependent (Fig. S5). All fitness ratios between all competitions were greater than a fitness ratio 1.00 and significant with two-tailed *t*-tests** (with type 1 α error=0.05), which had more statistical power than matched-paired samples and Mood’s tests [30, 32]. A common trend for each strain was for WS to have a higher competitive ability than SM when WS was the rarer variety. Thus, as the rare variety, WS seemed better able to invade than the SM ecotype (Fig. S5).

### Ecological diversification and biofilm evolution in the free-living stage increase microbial fitness in the squid host

Fig. 1 provides an overview of the squid colonization phenotypes commonly observed in *V. fischeri* wild isolates [41]. The solid brown line represents wild-type or ‘normal’ colonization in the sepiolid squid light organ by *V. fischeri *. The other coloured lines that are broken (i.e. dashed or discontinuous) represent deficiencies or defects in wild-type colonization. Most *V. fischeri* strains that are not indigenous symbionts from sepiolid squid will manifest a defect in host colonization [23, 41]. Wild-type colonization is characterized by an immediate squid infection (a short lag phase, usually no more than a few hours), bacterial persistence (a chronic infection) throughout the animal’s life, a short generation time during exponential growth and regrowth (~30 min), and a venting of no more than 90–95 % of the light organ bacteria [18]. When *V. fischeri* isolates are collected from sepiolid squid and then reintroduced into a hatchling squid host with an axenic light organ, the bacteria will display wild-type colonization [41]. Within a few hours after venting, the *V. fischeri* cells remaining in the squid regrow to full population size in the light organ. The maximal number of *V. fischeri* cells that is capable of occupying the light organ is dependent on the sepiolid squid species and the maturity of the animal [37].

**Fig. 3.**
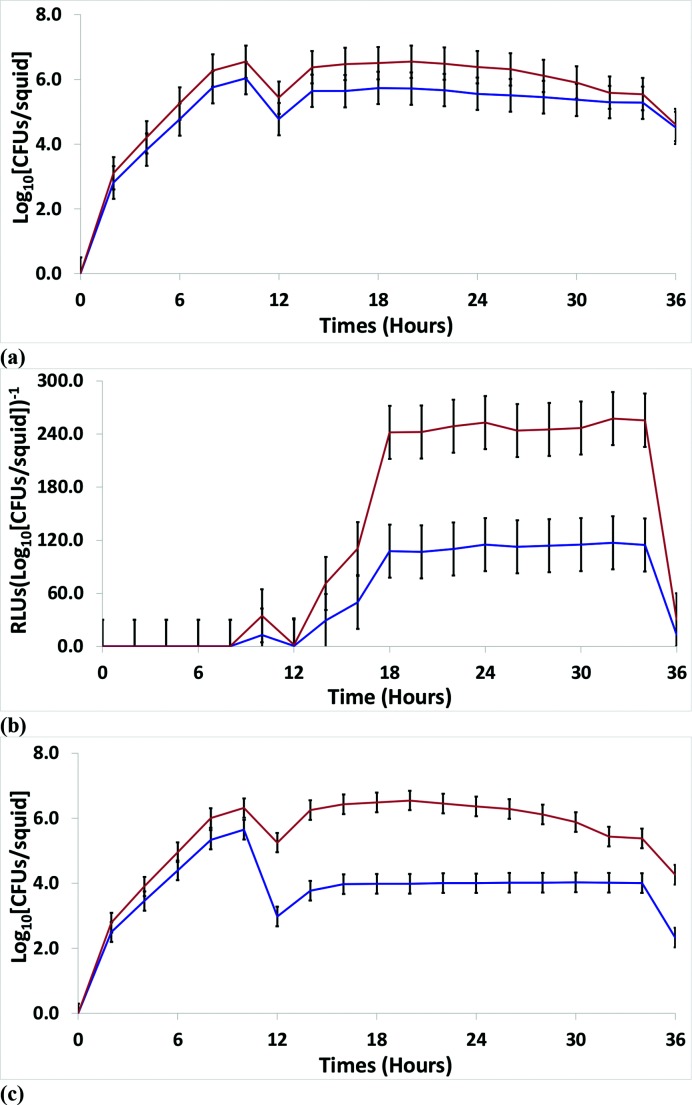
*In vivo* data for the group ‘all strains’. Using all 13 strains, monoculture experiments were performed with *V. fischeri* SM (blue) and WS (orange) ecotypes in *E. tasmanica* to examine squid colonization and growth (a) and bioluminescence [RLU per log_10_ (c.f.u. per squid)] (b). (c) Fifty/fifty competitions were conducted between SM (blue) and WS (orange) ecotypes in *E. tasmanica*. The error bars represent the least significant difference of the mean. RLU, relative light units.

**Fig. 4.**
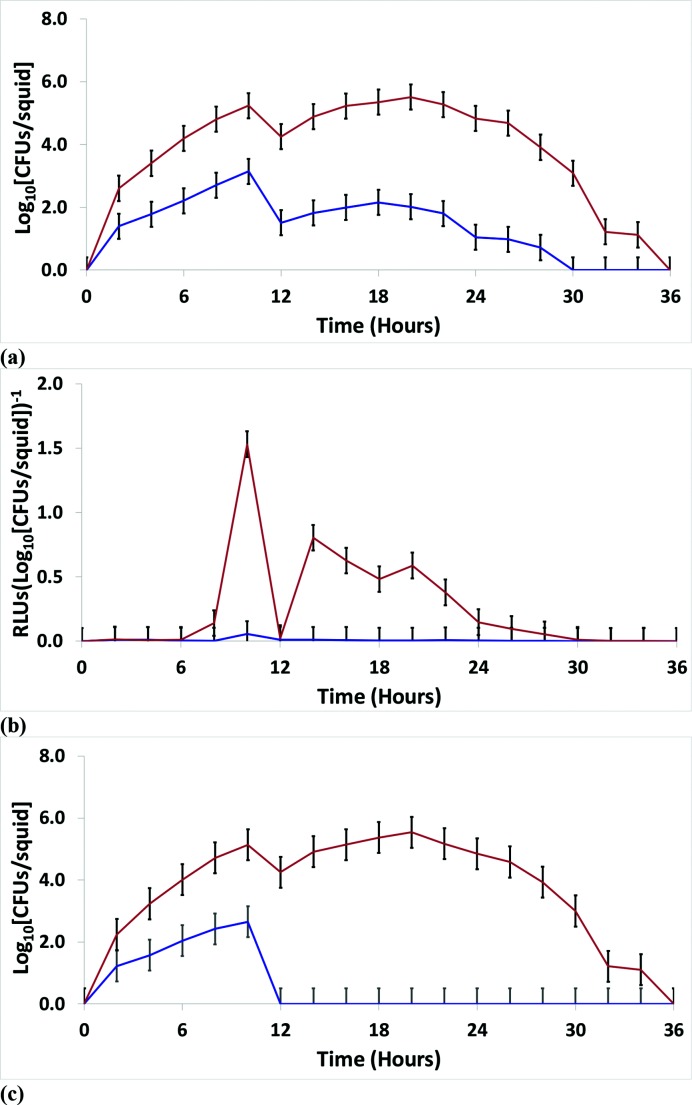
*In vivo* data for the group ‘free-living strains’. Using free-living strains of *V. fischeri* (see the text for the definition of free-living), monoculture experiments were performed with *V. fischeri* SM (blue) and WS (orange) ecotypes in *E. tasmanica* to examine squid colonization and growth (a) and bioluminescence [RLU per log_10_ {c.f.u. per squid)] (b). (c) Fifty/fifty competitions were conducted between SM (blue) and WS (orange) ecotypes in *E. tasmanica*. The error bars represent the least significant difference of the mean. RLU, relative light units.

**Fig. 5.**
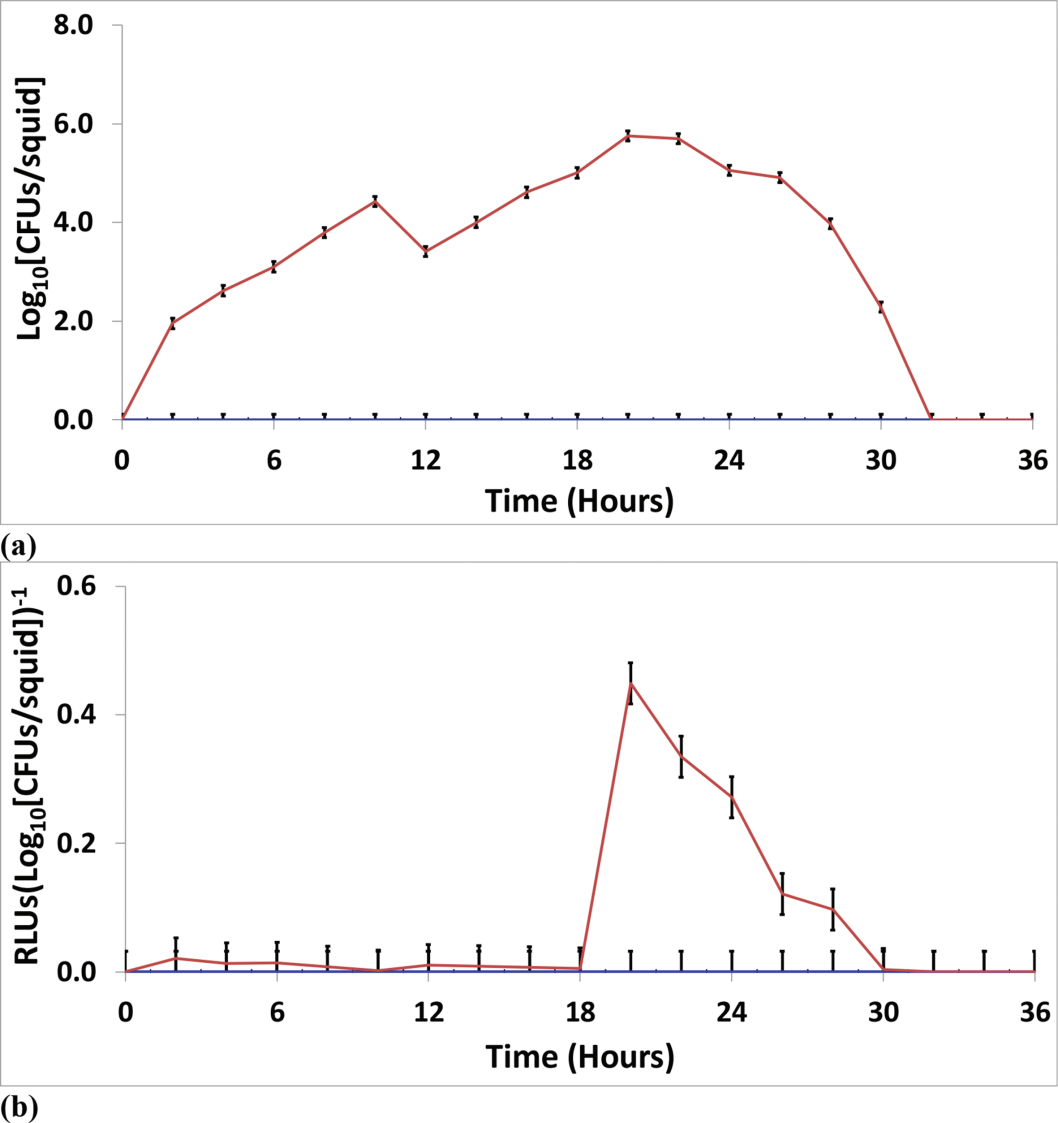
*In vivo* data for *V. fischeri* MDR7. Monoculture experiments were performed with *V. fischeri* MDR7 SM (blue) and WS (orange) ecotypes within *E. tasmanica* to examine squid colonization and growth (a) and bioluminescence [RLU per log_10_ (c.f.u. per squid)] (b). The error bars represent the least significant difference of the mean. RLU, relative light units.

Squid colonization (*in vivo*) data for *V. fischeri* are presented in Figs 3–5 and S6–S8. For all animal experiments, chloramphenicol resistance and kanamycin resistance were neutral markers (data not shown). These data are consistent with what has been reported previously [18, 25, 31]. Significant differences were documented in squid colonization and the resulting bioluminescence induced within the host between the ancestral SM ecotype and its WS derivative for all strains, yet the exact effect detected was sometimes strain-dependent (Figs 3–5 and S6–S8). For the *V. fischeri* strains originally isolated from sepiolid squid hosts (Table 1), the c.f.u. per squid host were identical between the SM and WS ecotypes when comparing monocultures in *E. tasmanica* (Figs S6a and S7a). For strains from *Euprymna* and *Sepiola*, *V. fischeri* SM and WS displayed wild-type colonization (solid brown line in Fig. 1) in the monoculture studies of *E. tasmanica*. In these pure culture experiments, the figures show that squid host colonization by the SM and WS ecotypes was synchronized and simultaneous, as the *in vivo* growth curves between the two ecotypes are seldom out of phase between replicate hosts (Figs S6a and S7a).

When including all strains in the analysis, there was a consistent trend for the derived WS ecotype to outperform the ancestral SM ecotype in *E. tasmanica* monocultures at all time points (Fig. 3a). The consistent trend of WS over SM in *E. tasmanica* monocultures was also observed in *V. fischeri* isolated from fish light organs (Fig. S8a). In a similar analysis encompassing all free-living strains, the derived WS ecotype significantly surpassed the SM ecotype at all time points in *E. tasmanica* monocultures (Fig. 4a). *V. fischeri* isolates indigenous to monocentrid fish light organs (Fig. S8) manifested deficiencies in numerous aspects of the wild-type squid colonization phenotype (solid brown line in Fig. 1). The deficiencies included a lower stationary phase, slower growth upon initial colonization, slower regrowth after venting and acute infection (broken orange, purple, grey and black lines in Fig. 1). Free-living strains of *V. fischeri* restricted to exist as saprophytes, marine sediment microbiota, bacterioplankton and commensals associated with marine animals also manifested deficiencies in the wild-type colonization of the squid host (Fig. 4). Free-living strain *V. fischeri* MDR7 was especially defective, as the SM ecotype of this strain completely failed to colonize the squid host (Fig. 5, broken blue line in Fig. 1). See below for more details on *V. fischeri* MDR7. For all strains, the derived *V. fischeri* WS ecotype outcompeted the ancestral *V. fischeri* SM within the squid host during 50 : 50 competitions (Figs 3c , 4c and S6c, S7c, S8c). This was also true when all 13 strains were analysed individually for 50 : 50 competitions in *E. tasmanica* (data not shown).

The squid colonization data for the WS ecotype was especially interesting for one particular strain, namely *V. fischeri* MDR7 (Fig. 5). *V. fischeri* MDR7 WS colonized *E. tasmanica* in monoculture, while *V. fischeri* MDR7 SM did not (Fig. 5a). The result for the 50 : 50 competition between *V. fischeri* MDR7 WS and *V. fischeri* MDR7 SM is not illustrated but was identical to the monoculture result. That is, *V. fischeri* MDR7 WS colonized the squid host, while *V. fischeri* MDR7 SM did not (Fig. 5a). While *V. fischeri* MDR7 SM was completely incapable of initiating squid host colonization, *V. fischeri* MDR7 WS had an ephemeral residence in the squid light organ (acute infection), essentially producing a host range expansion in *V. fischeri* MDR7 from an obligately free-living strain to an ‘acute’ symbiont. Thus, a ‘host’ transition occurred from the broken blue line to a broken black line in Fig. 1 due to a niche shift (‘planktonic’ to ‘pellicle’) in the free-living stage of *V. fischeri* MDR7. The *in vivo* data for the other 12 strains were also analysed individually, but their results are not shown to conserve space. For all groups of strains, squid colonized by pure cultures of the descendent *V. fischeri* WS ecotype were more bioluminescent than animal hosts colonized by the *V. fischeri* SM ancestor (Figs 3b, 4b, 5b and S6b, S7b, S8b). This was also true when all 13 strains were analysed individually for monocultural bioluminescence in *E. tasmanica* (data not shown except for *V. fischeri* MDR7 Fig. 5b).

## Discussion

Horizontal gene transfer during the free-living phase has long been known to impact on the evolution of host–microbe interactions and host range in *Vibrio *; however, the immediate effects of novel mutations (i.e. microevolution) have been less clear [42]. The deliberation of horizontal gene transfer versus mutation in influencing the evolution of host range in *Vibrio* is analogous to the classical discussion of antigenic shift versus antigenic drift with influenza virus [43]. For all *V. fischeri* strains examined, this study demonstrated that novel genetic changes during the free-living phase had drastic and rapid effects on host–microbe interactions. This was especially true for the free-living strains. For example, compare the WS and SM ecotypes in the monoculture studies and the 50 : 50 competitions in Fig. 4a–c. The results with *V. fischeri* MDR7 were extraordinary. *V. fischeri* MDR7 SM, which could not colonize *E. tasmanica* at all, underwent host range expansion as a result of evolution outside the host environment (Fig. 5).

Additionally, the ancestral correlation between SM motility and SM biofilm formation, which was a significant negative linear relationship, changed drastically as a result of ecological diversification in static liquid microcosms (Fig. 2). A negative association (antagonistic pleiotropy) between motility and biofilm formation has frequently been reported in bacterial physiology research [44]. One possible explanation is that there is a metabolic constraint inherent to intracellular c-di-GMP regulation. The roles of c-di-GMP, motility, biofilm formation and exopolymer synthesis (e.g. cellulose production) in the squid–* Vibrio* symbiosis have been extensively reviewed [45–47]. Nevertheless, the negative correlation between motility and biofilm formation was reduced in the derived *V. fischeri* WS ecotype as a result of evolution occurring outside a light organ host. Thus, negative correlations which are caused by complex antagonistic pleiotropy and gene interactions (such as in motility and biofilm formation) are themselves traits that can evolve [10, 48]. Inverse correlations and antagonistic pleiotropy can limit or slow microbial adaptation to host–microbe interactions due to the fitness tradeoffs that may result. The data in Fig. 2 exemplify a proof a principle. Namely, evolution during the free-living stage has the potential to erode the fitness tradeoffs imposed by negatively correlated traits [49]. Numerous mechanisms exist to account for how the erosion of tradeoffs may proceed [9, 10, 48]. Antagonistic pleiotropy itself is a variable character that can be a target of selection [50, 51]. The ancestral SM tradeoff between motility and biofilm may have been an evolutionary constraint for further squid host adaptation by *V. fischeri* populations evolving in a host-adaptive landscape [52, 53]. Motility and biofilm proficiency have both been shown to be beneficial traits for colonizing animal hosts, including squid [54]. Consequently, microbial evolution during the free-living phase possibly opened up further adaptive possibilities or made available alternative evolutionary trajectories for improving fitness in a natural host by easing the physiological constraint between two traits known to be useful for colonizing animals [52].

The change in correlation between motility and biofilm formation was caused by mutations in genetically distinct strains (Fig. 2). This result was consistent with differential epistasis, which describes how antagonistic pleiotropy can change due to the evolution of gene interactions in a genomic background [49]. Although antagonistic pleiotropy has frequently been seen as an evolutionary constraint, prior work has also shown that antagonistic pleiotropy can drive genetic innovation and adaptive evolution [55, 56]. When considered as a polymorphic character, pleiotropy can increase a populations’s ability to respond successfully to new and challenging selection pressures [57]. Additionally, pleiotropy can create adaptive landscapes with rugged topographies, where the fitness peaks have different heights [58]. For microbial populations constantly experiencing systematic changes in the environment due to fluctuations between free-living and host-associated lifestyles, antagonistic pleiotropy can mobilize peak shifts to progressively higher fitness optima in a host-adaptive landscape [59–61]. The host range expansion in *V. fischeri* MDR7 was an example of a dramatic peak shift to a higher fitness optimum (Fig. 5). When the fitness peaks are in constant motion as a result of fluctuating adaptive landscapes, which is likely in cyclical free-living and host environments, tracking fitness peaks that are moving targets becomes an especially important property of pleiotropy evolution (see the flying-kite and diving-kite effects in [59]).

Positive pleiotropy was responsible for microbial fitness during the free-living and host-associated stages. Microbial positive pleiotropy has been identified in carbon source utilization, cheater control and cross-protection against multiple environmental stressors [12, 62, 63]. Positive pleiotropy may be expected for genes affecting fitness traits that need to be maximized or maintained efficiently across varying life histories (e.g. free-living versus host environments), especially if all the alternative life history stages occur in stressful environments or during episodes when resources are scarce [64]. During the free-living stage, microbes contend with starvation and extreme abiotic factors. In the host environment, micro-organisms confront tenacious immune defences [65].

Positive pleiotropy can elevate the capacity for adaptive evolution by enabling phenotype integration [66]. When shaping microbial fitness, natural selection could be operating on cyclical free-living and host environments in a manner analogous to a life cycle, life history, or ontogeny, where various parameters and fitness components (reproduction, survival, rate of horizontal gene transfer, dispersal to new hosts, etc.) are weighed differently at each life stage [67–69]. Under such circumstances, trait covariation ensures that adaptive character changes acquired directly in the free-living stage (due to positive selection) are accompanied by indirect alterations in other phenotypes, which accommodate and complement host colonization [66, 70]. For example, elevated tolerance to nutrient starvation in the free-living stage is correlated with increased intracellular survival in macrophages (phagocytic immune cells) by some human bacterial pathogens [71, 72].

In prokaryotes, the genetic networks that cross-talk between motility and biofilm formation possess modular pleiotropic properties [73–75]. Modular pleiotropy is especially associated with increased rates of adaptation and faster population recovery, since a larger proportion of beneficial mutations with large effects are substituted more quickly in gene pools [76]. Modular pleiotropy is also positively correlated with evolvability, robustness and horizontal gene transfer [49, 77, 78]. Modular pleiotropy has been hypothesized to mediate phenotype integration and trait specialization across different life history stages [79].

The *V. fischeri* WS ecotype showed greater biofilm formation (Fig. S4a) and brighter bioluminescence than the SM ancestor (Figs 3b, 4b, 5b and S6b, S7b, S8b). Increased biofilm capacity and raised bioluminescence levels have previously been shown to be favourable traits in *V. fischeri* for colonizing the squid host *E. tasmanica* [18, 25]. In the ocean and marine habitats, an increased biofilm capacity by *V. fischeri* can facilitate attachment to suspended particulate matter or marine sediment, where nutrients might be concentrated through adsorption on abiotic surfaces [80–82]. Environmental gradients will form along surfaces such as sediment, suspended debris, marine snow and detritus. These environmental gradients will foster bacterial aggregations and biofilms at liquid–solid interfaces, which in turn will drive the ecological diversification and biofilm evolution that can facilitate host–microbe interactions [39, 83, 84].

Biofilms also limit the deleterious effects of environmental stressors by providing gradients, where different levels of tolerance of microbes to the stressors are possible (e.g. a continuum of increasing oxidative stress) [85]. These stressor gradients provide stepping stones that permit mutations of smaller beneficial effect to be established in the biofilm population as a result of natural selection. Moreover, biofilms can drastically increase the mutation rates of bacteria [85]. Along with a heightened tolerance to environmental stressors, biofilms also provide bacterial cells with increased metabolic efficiency and extra defence against antimicrobial agents [81]. The pellicle tensile strength data (Fig. S3) demonstrate clearly that biofilm evolution in *V. fischeri* can lead to ecosystem engineering and niche construction [38, 84].

The evolutionary origin of bioluminescence is an enigma, since this trait’s emergence in bacteria predated animals with visual sensory systems [86]. Bioluminescence during the free-living phase has been proposed to be a strategy against oxidative stress and reactive oxygen species [65]. It has also been suggested that bioluminescence is a DNA repair mechanism that bacteria can continue to utilize at night-time when outside their animal hosts [87]. Moreover, bioluminescence has been hypothesized to drive chemical reactions, where trace oxygen and reactive oxygen species could be scavenged as electron acceptors at severely low oxygen concentrations, permitting the catabolism of carbon sources for reducing power [86]. The electron transport pathway mediated by cytochrome oxidase is inhibited at extremely low oxygen levels [88]. In summary, for all *V. fischeri* strains examined in this study, microbial evolution via niche adaptation while away from the host environment apparently generated exaptations (increased bioluminescence and elevated biofilm formation) that were repurposed for improved host colonization.

Niche invasion and ecological diversification in the static liquid microcosms resulted from negative frequency-dependent selection (Fig. S5), which generated and maintained diversity (Fig. S1a and S1b). Since the rare ecotype was always invaded, there is no direct evidence to suggest that microbial allelopathy is occurring between the SM and WS ecotypes in the squid host. However, negative frequency-dependent selection *in vitro* does not necessarily preclude the possibility of microbial biochemical warfare in the host light organ. Environmental heterogeneity, spatial structure and ecological opportunity (vacant niches) were necessary for ecological diversification, as evolutionary differentiation failed to occur in the disturbed cultures (Fig. S1c). Moreover, this study demonstrated that the deterioration of host colonization by a microbe is not always a mandatory outcome of evolution occurring outside the host environment. Most bacterial cells in nature are believed to exist as biofilms [81], and biofilms growing in spatially structured environments undergo genetic and ecological diversification [89]. Even in the absence of horizontal gene transfer, biofilm evolution occurring extrinsically to host environments rapidly promoted animal host colonization and broadened host range through novel yet simple mutations. Thus, microbial biofilm evolution during the free-living stage may be more common than has previously been appreciated in facilitating symbioses and influencing host–microbe interactions.

## Supplementary Data

Supplementary File 1
