# Supplementary material for: Symbiont evolution during the free-living phase can improve host colonization
Source: Microbiology (Reading). 2019 Jan 16;165(2):174–87. doi: 10.1099/mic.0.000756 (PMC7003651; doi:10.1099/mic.0.000756)
Supplement: Supplementary File 1 [file mic-165-174-s001.pdf]

SUPPLEMENTARY MATERIAL

Motivation for Using Static Liquid Microcosms

The first goal was to simulate microbial evolutionary processes—exploitation of ecological opportunity, invasion of empty niches, ecological diversification, and biofilm evolution—that microbial symbionts, pathogens, and commensals are known to experience in nature when outside their host environments [1]. The second goal was to see the impact of these evolutionary processes on host colonization, especially when biofilm evolution is known to strongly influence microbial ecological diversification and host-microbe interactions [1]. How microbial exploitation of ecological opportunity (*e.g.*, vacant niches) in the free-living phase influences host-microbe associations has not been previously examined. Microorganisms live in habitats where environmental heterogeneity (*e.g.*, gradients of resources and abiotic factors) prevails on a fine-scale to produce micro-niches measured in micrometers [2]. In nature, these micro-niches typically possess spatial structure, which microbes invade as biofilms on surfaces and liquid-air interfaces [3]. Spatial structure and biofilm evolution are drivers of adaptive radiation, niche construction, and ecotype formation in microorganisms [1, 4, 5]. Increased biofilm formation and ecological diversification have both been positively correlated with greater colony phenotype diversity [6]. Vibrionaceae are renown for two characteristics, forming biofilms in the free-living stage and entering host-microbe associations [7]. Moreover, changes in colony morphology are known to impact how the Vibrionaceae interact with their hosts [7]. In summary, ecological diversification in static liquid microcosms was used to model evolution during the free-living stage, since derived colony variants with greater biofilm capacity than the ancestor were hypothesized to be positively pleiotropic for higher microbial fitness in the host environment [8].

The use of static liquid microcosms for this study is justified, since it is consistent with the evolutionary ecology of the Vibrionaceae [9-11].

Supplementary Figure Legends

Fig. S1 Ecological diversification in static microcosms, relative diversity, and nonculturability in shaking microcosms. (a) Cell densities were calculated for *V. fischeri* SM (blue) and WS (orange) in static liquid microcosms over 22 days for all strains. **(b)** Relative diversity (*J'*) was calculated for *V. fischeri* in static liquid microcosms over 22 days for all strains. Cell densities were calculated for *V. fischeri* in shaking test tube cultures, but liquid cultures became completely nonculturable on agar plates within two or three days, erasing the opportunity for characterizing the origins of ecological diversity (the onset of polymorphisms in SM and WS). **(c)** For brevity, the "shaking test tube" data is only shown for *V. fischeri* WH4. However, similar results were obtained for all *V. fischeri* strains. Error bars represent standard error of the mean.

Fig. S2 SM and WS morphotypes are each distinct ecotypes. An SM colony at **(a)** 24 hours and 35X **(b)** 48 hours and 15X, and **(c)** 72 hours and 15X as seen through a stereo microscope (28ºC incubation). A WS colony at **(d)** 24 hours and 35X, **(e)** 48 hours and 15X, and **(f)** 72 hours and 15X as seen through a stereo microscope (28ºC incubation). When SM and WS colonies were streaked for isolation, each only produced SM and WS colonies, respectively. Scale bar = 100 µM in (a) & (d) and 400 µM in (b), (c), (e) & (f). **(g)** Non-shaking liquid cultures produced by *V. fischeri* WS (left) and SM (right) morphotypes as seen from above at a slanting view.

Fig. S3 Using glass beads to quantify pellicle tensile strength. (a) A test tube was inverted that contained a liquid culture with a pellicle 5-days old. The ceiling lights and the flask with the blue liquid in the foreground provides perspective, demonstrating the test tube is truly upside down. **(b)** The pellicle is clearly seen in this magnified image. **(c)** Glass beads were used to quantify the tensile strengths of pellicles. **(d)** Breaking a 5-day old pellicle was not possible with a full column or stack of glass beads in the test tube. **(e)** For all strains, the *V. fischeri* WS (orange) ecotype possessed higher tensile strength than the *V. fischeri* SM (blue) ecotype. Error bars represent least significant difference of the mean.

Fig. S4 Biofilm and motility in the SM and WS ecotypes. Biofilm assays **(a)** and motility assays **(b)** were conducted with the *V. fischeri* WS (orange) and SM (blue) ecotypes for each strain. In the biofilm assay, the black bar represents the negative control. **(c)** There was a significant positive linear relationship between pellicle tensile strength and biofilm formation. For (a) and (b), error bars represent least significant difference of the mean. For (c), error bars represent standard error of the mean.

Fig. S5 Invasion experiments examining negative frequency-dependent selection between SM and WS. Competitive invasion experiments were done between the SM and WS ecotypes for all *V. fischeri* strains over two weeks. Arrows point from the invading ecotype (originally

rare) to the invaded variety (originally common), where the starting ratio for the two competitors was 100:1. Numbers along each line were calculated fitness ratios for each experiment.

Fig. S6 *In vivo* data for the group "*Euprymna* strains". Using strains isolated from *Euprymna* spp., monoculture experiments were done with *V. fischeri* SM (blue) and WS (orange) ecotypes in *E. tasmanica* examining squid colonization and growth **(a)** and bioluminescence (RLUs per $\log_{10}$[CFUs per squid]) **(b)**. 50:50 competitions were conducted between SM (blue) and WS (orange) ecotypes **(c)** in *E. tasmanica*. Error bars represent least significant difference of the mean. RLUs = relative light units.

Fig. S7 *In vivo* data for the group "*Sepiola* strains".Using strains isolated from *Sepiola* spp., monoculture experiments were done with *V. fischeri* SM (blue) and WS (orange) ecotypes in *E. tasmanica* examining squid colonization and growth **(a)** and bioluminescence (RLUs per $\log_{10}$[CFUs per squid]) **(b)**. 50:50 competitions were conducted between SM (blue) and WS (orange) ecotypes **(c)** in *E. tasmanica*. Error bars represent least significant difference of the mean. RLUs = relative light units.

Fig. S8 *In vivo* data for the group "fish strains".Using strains isolated from fish light organs, monoculture experiments were done with *V. fischeri* SM (blue) and WS (orange) ecotypes in *E. tasmanica* examining squid colonization and growth **(a)** and bioluminescence (RLUs per $\log_{10}$[CFUs per squid]) **(b)**. 50:50 competitions were conducted between SM (blue) and WS (orange) ecotypes **(c)** in *E. tasmanica*. Error bars represent least significant difference of the mean. RLUs = relative light units.

Supplementary Figures

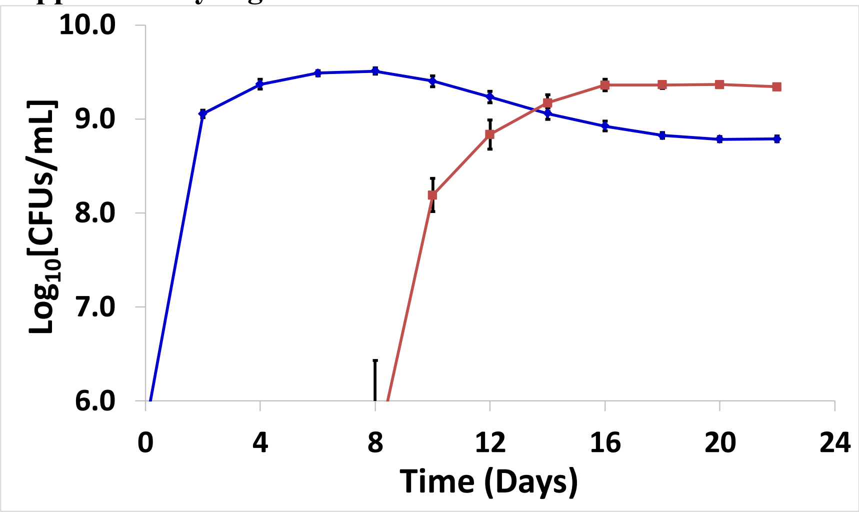

(a)

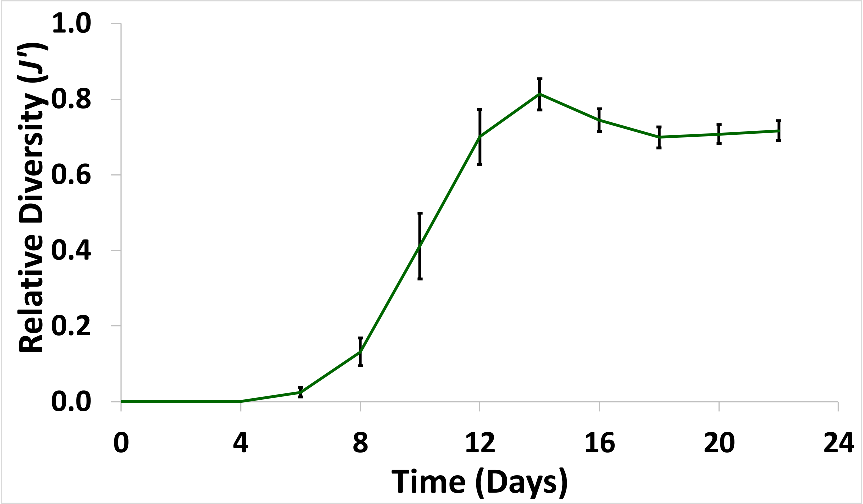
Relative Diversity (J')
1.0
0.8
0.6
0.4
0.2
0.0
0
4
8
12
16
20
24
Time (Days)

(b)

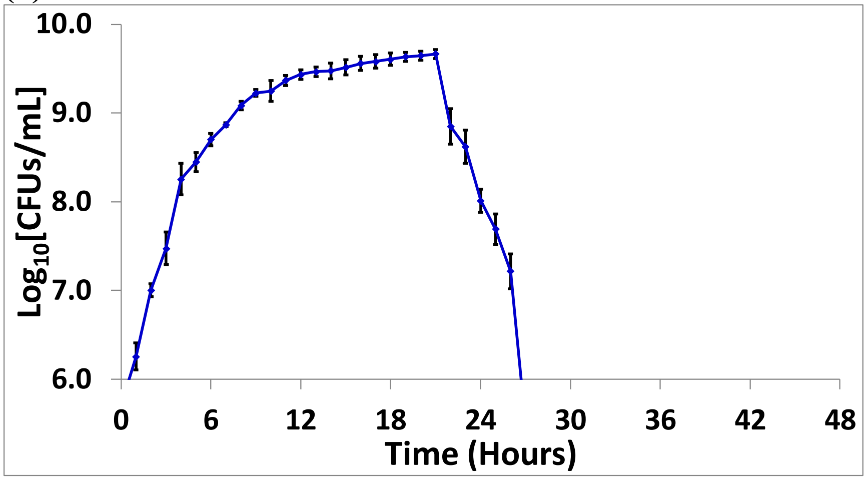
Log_{10}[CFUs/mL]
10.0
9.0
8.0
7.0
6.0
0
6
12
18
24
30
36
42
48
Time (Hours)

(c)

Fig. S1

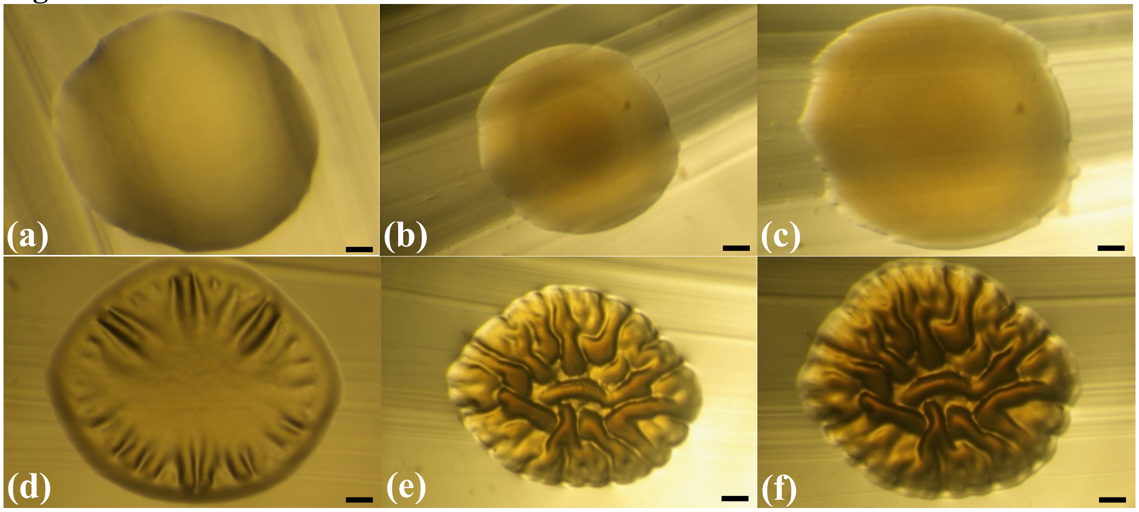
(a)
(b)
(c)
(d)
(e)
(f)

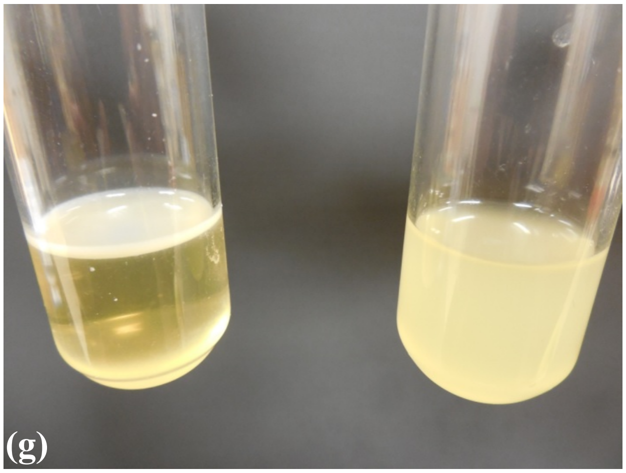

Fig. S2

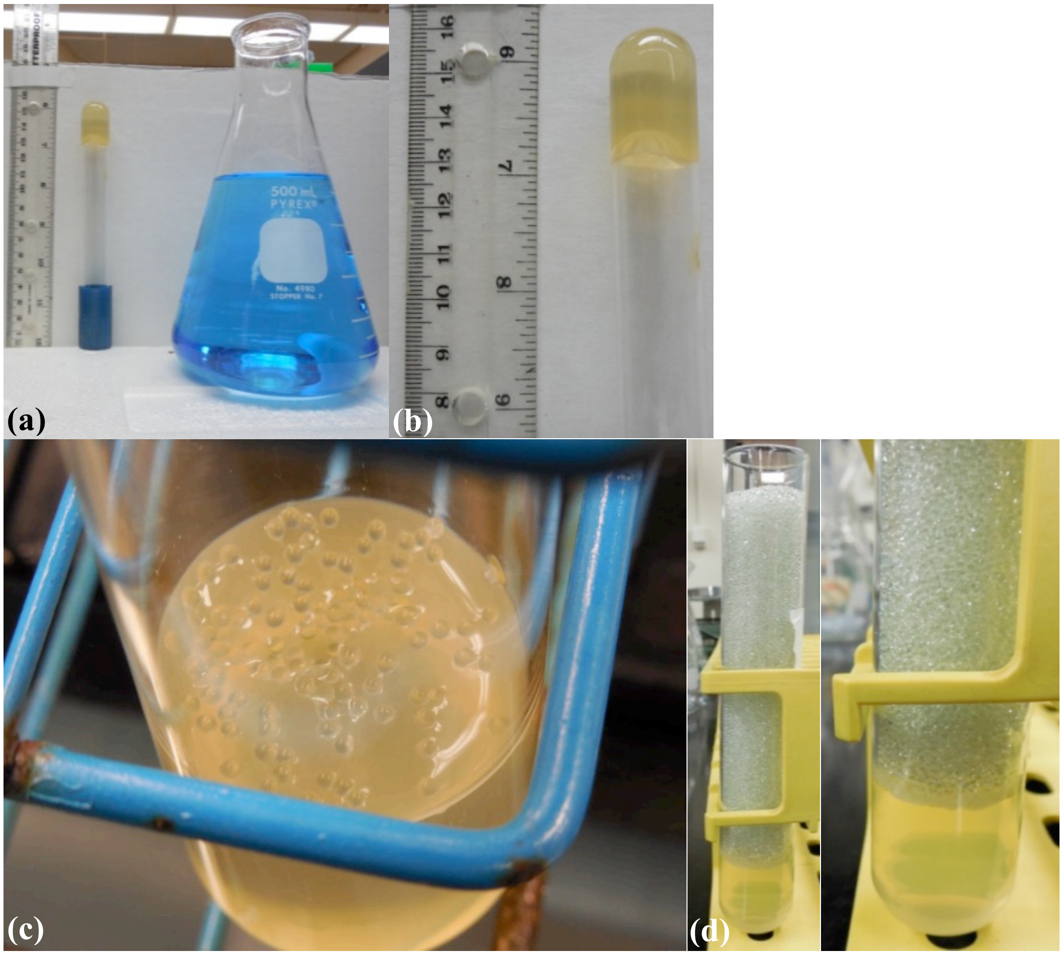

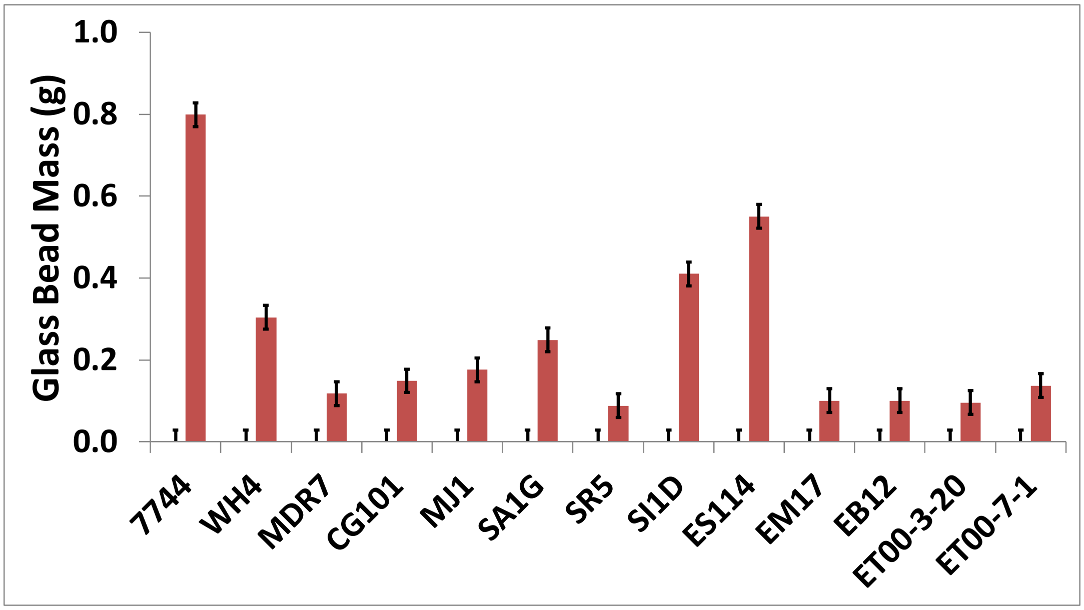
Glass Bead Mass (g)
1.0
0.8
0.6
0.4
0.2
0.0
7744
WH4
MDR7
CG101
MJ1
SA1G
SR5
SI1D
ES114
EM17
EB12
ET00-3-20
ET00-7-1

(e)

Fig. S3

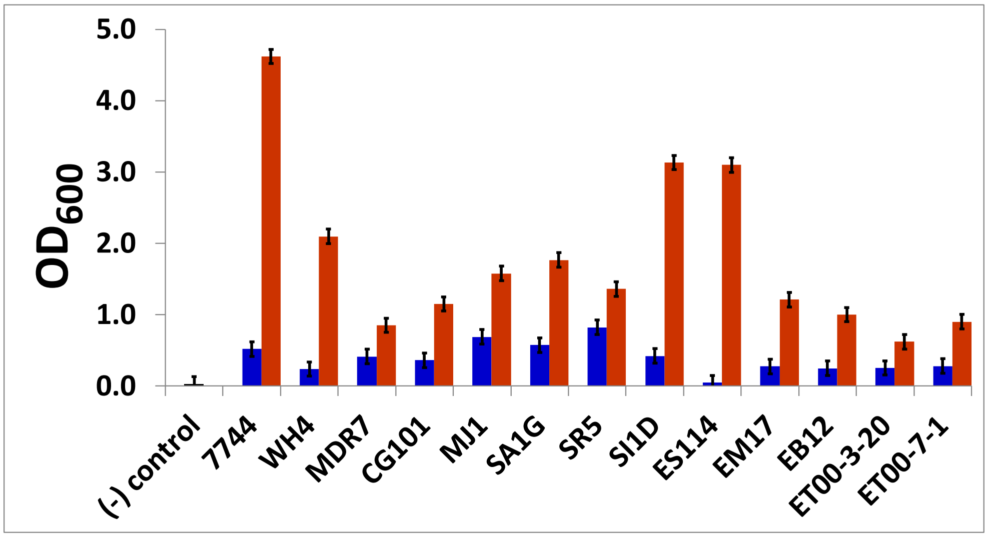
OD_{600}
5.0
4.0
3.0
2.0
1.0
0.0
(-) control
7744
WH4
MDR7
CG101
MJ1
SA1G
SR5
SI1D
ES114
EM17
EB12
ET00-3-20
ET00-7-1

(a)

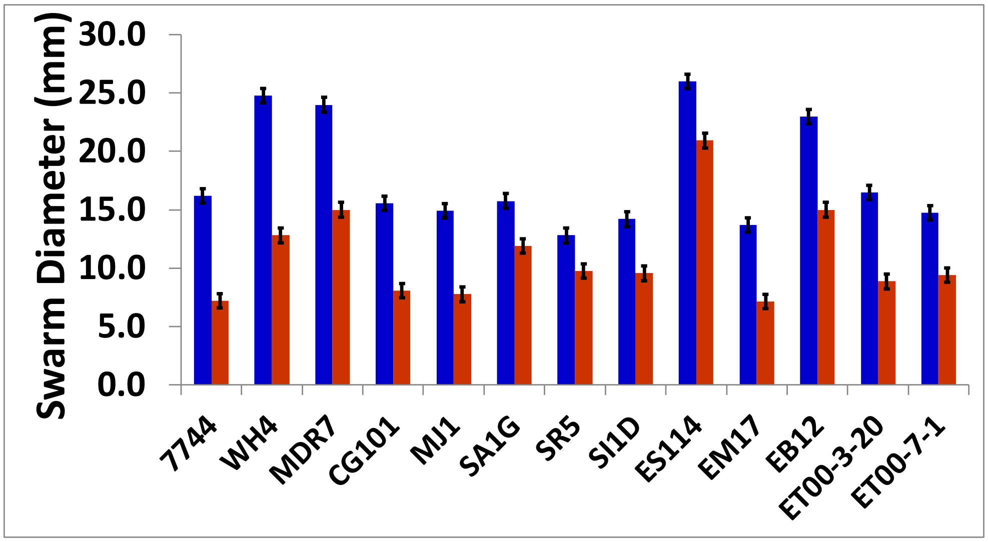

Swarm Diameter (mm)
30.0
25.0
20.0
15.0
10.0
5.0
0.0
7744
WH4
MDR7
CG101
MJ1
SA1G
SR5
SI1D
ES114
EM17
EB12
ET00-3-20
ET00-7-1

(b)

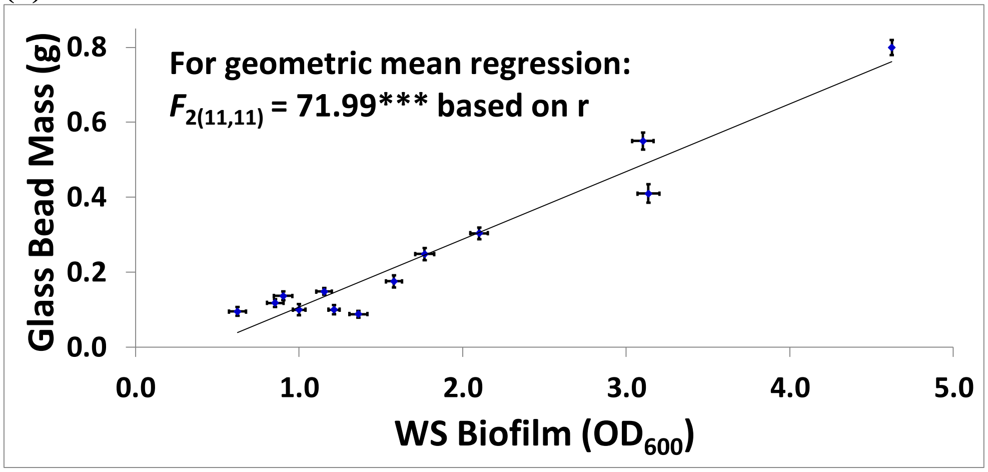

For geometric mean regression:
$F_{2(11,11)}$ = 71.99*** based on r
Glass Bead Mass (g)
0.8
0.6
0.4
0.2
0.0
0.0
1.0
2.0
3.0
4.0
5.0
WS Biofilm (OD_{600})

(c)

Fig. S4

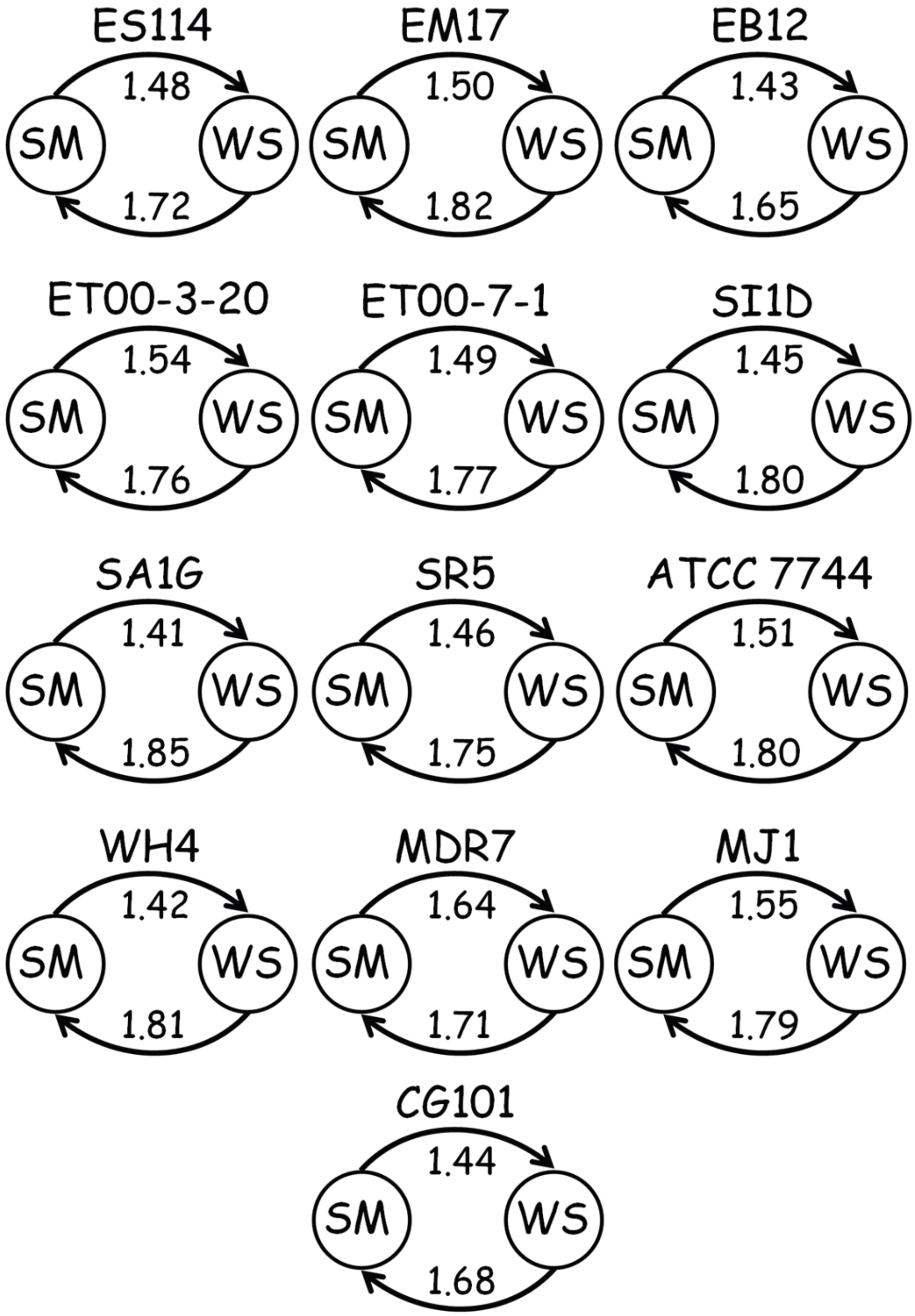
ES114
1.48
SM
WS
1.72
EM17
1.50
SM
WS
1.82
EB12
1.43
SM
WS
1.65
ET00-3-20
1.54
SM
WS
1.76
ET00-7-1
1.49
SM
WS
1.77
SI1D
1.45
SM
WS
1.80
SA1G
1.41
SM
WS
1.85
SR5
1.46
SM
WS
1.75
ATCC 7744
1.51
SM
WS
1.80
WH4
1.42
SM
WS
1.81
MDR7
1.64
SM
WS
1.71
MJ1
1.55
SM
WS
1.79
CG101
1.44
SM
WS
1.68

Fig. S5

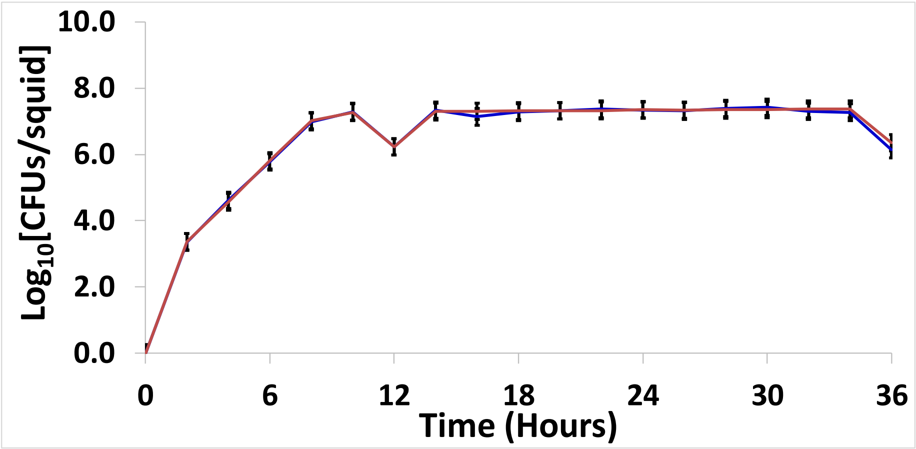

Log$_{10}$[CFUs/squid]
10.0
8.0
6.0
4.0
2.0
0.0
0
6
12
18
24
30
36
Time (Hours)

(a)

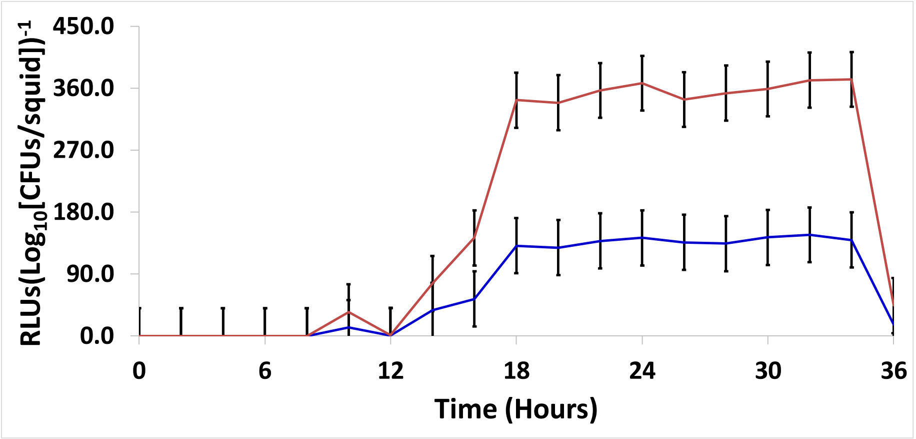

RLUs(Log$_{10}$[CFUs/squid])$^{-1}$
450.0
360.0
270.0
180.0
90.0
0.0
0
6
12
18
24
30
36
Time (Hours)

(b)

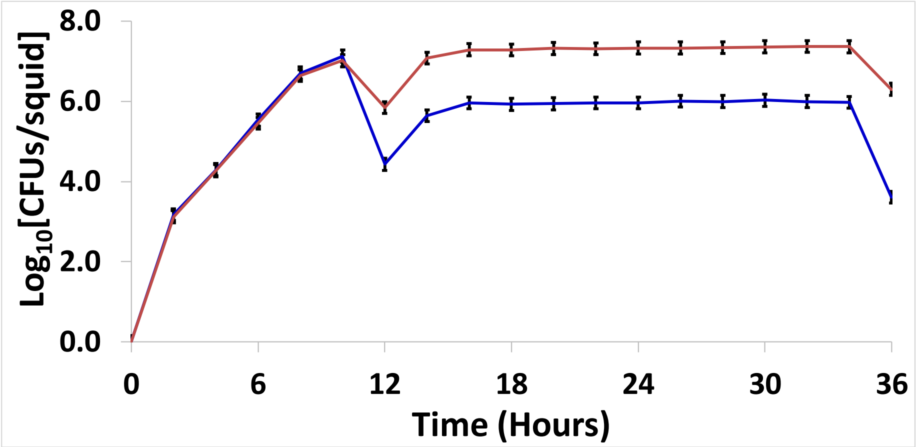

Log$_{10}$[CFUs/squid]
8.0
6.0
4.0
2.0
0.0
0
6
12
18
24
30
36
Time (Hours)

(c)

Fig. S6

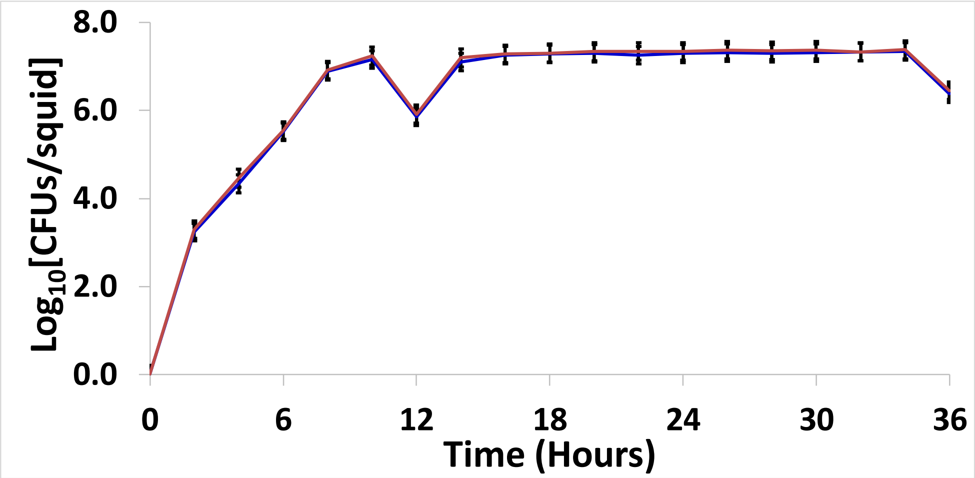
Log10[CFUs/squid]
8.0
6.0
4.0
2.0
0.0
0
6
12
18
24
30
36
Time (Hours)

(a)

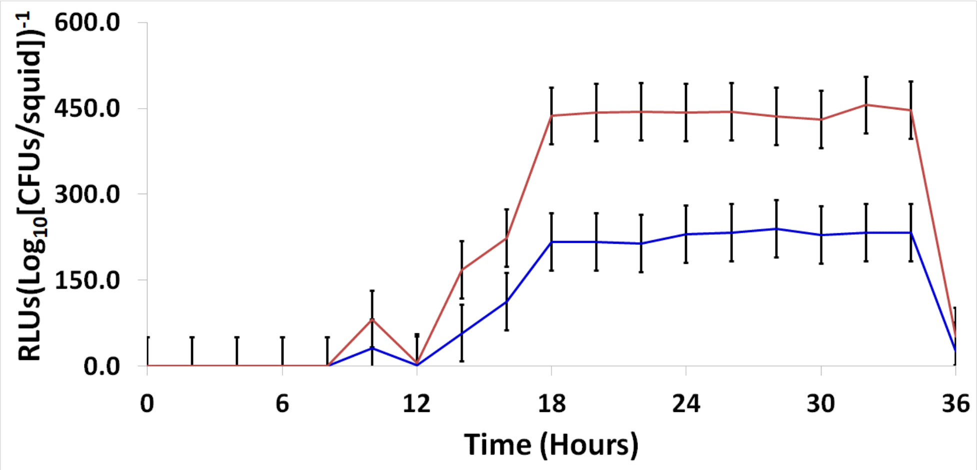
RLUs(Log10[CFUs/squid])-1
600.0
450.0
300.0
150.0
0.0
0
6
12
18
24
30
36
Time (Hours)

(b)

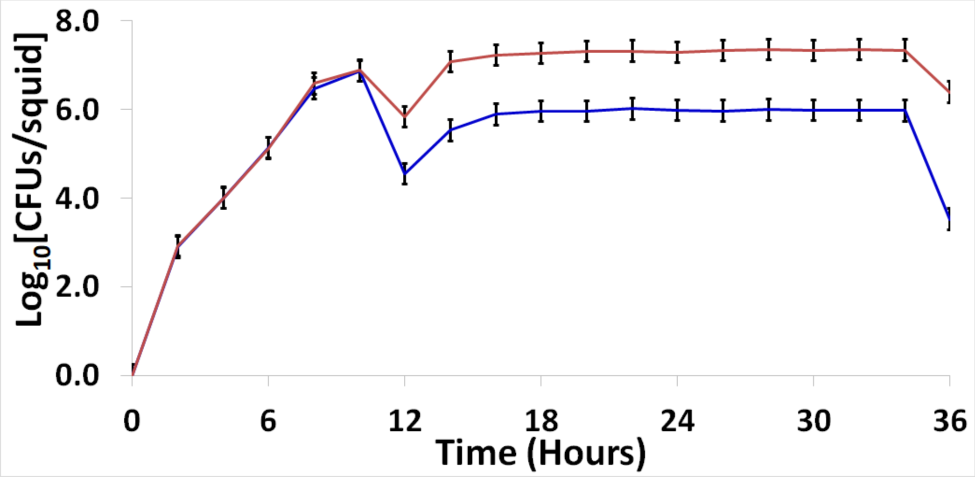
Log10[CFUs/squid]
8.0
6.0
4.0
2.0
0.0
0
6
12
18
24
30
36
Time (Hours)

(c)

Fig. S7

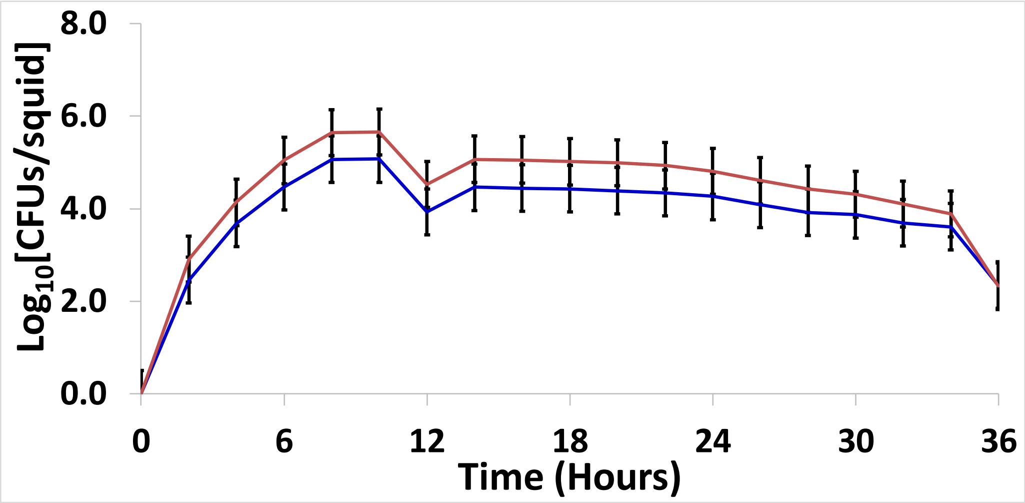

8.0
6.0
4.0
2.0
0.0
Log10[CFUs/squid]
0
6
12
18
24
30
36
Time (Hours)

(a)

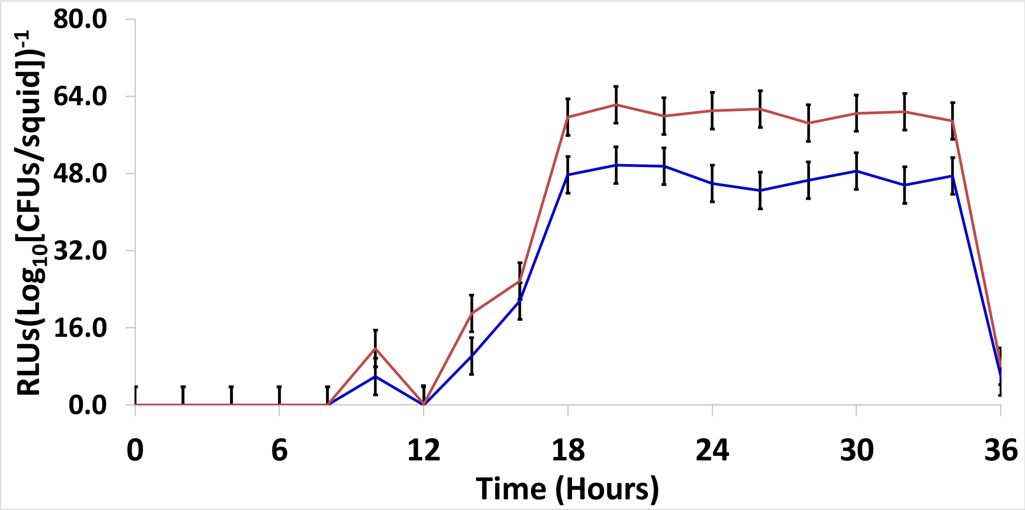

80.0
64.0
48.0
32.0
16.0
0.0
RLUs(Log10[CFUs/squid])-1
0
6
12
18
24
30
36
Time (Hours)

(b)

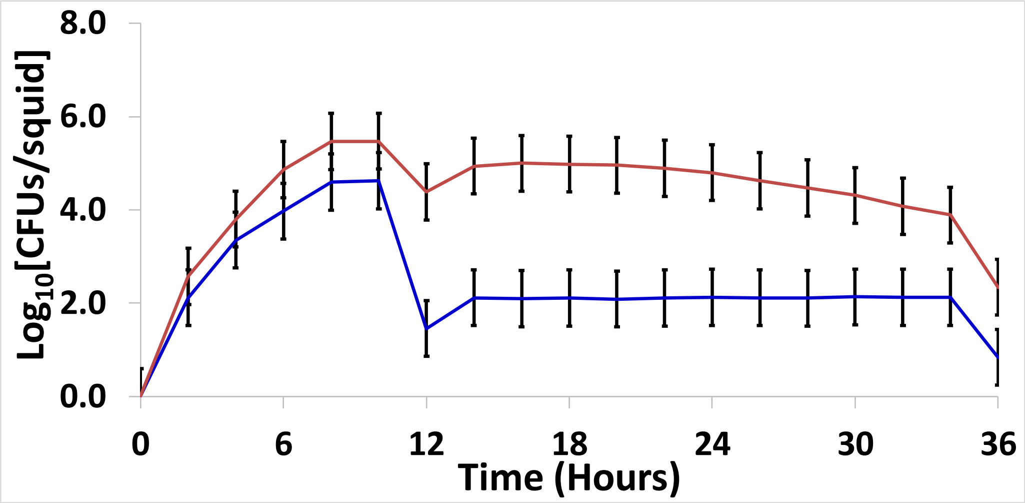

8.0
6.0
4.0
2.0
0.0
Log10[CFUs/squid]
0
6
12
18
24
30
36
Time (Hours)

(c)

Fig. S8

SUPPLEMENTARY REFERENCES

1. **Steenackers HP, Parijs I, Foster KR, Vanderleyden J**. Experimental evolution in biofilm populations. *FEMS Microbiology Reviews* 2016;40:373–397.

2. **Korona R, Nakatsu CH, Forney LJ, Lenski RE**. Evidence for multiple adaptive peaks from populations of bacteria evolving in a structured habitat. *Proceedings of the National Academy of Sciences* 1994;91:9037-9041.

3. **Ponciano JM, La H-J, Joyce P, Forney LJ**. Evolution of diversity in spatially structured *Escherichia coli* populations. *Applied and Environmental Microbiology* 2009;75:6047–6054.

4. **Eastman JM, Harmon IJ, La H-J, Joyce P, Forney IJ**. The onion model, a simple neutral model for the evolution of diversity in bacterial biofilms. *Journal of Evolutionary Biology* 2011;24:2496–2504.

5. **Koza A, Moshynets O, Otten W, Spiers AJ**. Environmental modification and niche construction: developing O2 gradients drive the evolution of the wrinkly spreader. *International Society for Microbial Ecology Journal* 2011;5:665–673.

6. **Rainey PB, Travisano M**. Adaptive radiation in a heterogeneous environment. *Nature* 1998;394:69-72.

7. **Soto W, Nishiguchi MK**. Microbial Experimental Evolution as a Novel Research Approach in the Vibrionaceae and Squid-*Vibrio* Symbiosis. *Frontiers in Microbiology doi:103389/fmicb201400593* 2014.

8. **Soto W, Rivera FM, Nishiguchi MK**. Ecological diversification of *Vibrio fischeri* serially passaged for 500 generations in novel squid host *Euprymna tasmanica*. *Microbial Ecology* 2014;67:700-721.

9. **Thompson JR, Polz MF**. Dynamics of *Vibrio* populations and their role in environmental nutrient cycling. In: Thompson FL, Austin B, Swings J (editors). *The Biology of Vibrios*. Washington, D.C.: ASM Press; 2006.

10. **Urakawa H, Rivera ING**. Aquatic environments. In: Thompson FL, Austin B, Swings J (editors). *Biology of Vibrios*. Washington, D.C.: ASM Press; 2006.

11. **McDougald D, Kjelleberg S**. Adaptive responses of vibrios. In: Thompson FL, Austin B, Swings J (editors). *The Biology of Vibrios*. Washington, D.C.: ASM Press; 2006.
